# Hospital Sunday Fund

**Published:** 1903-06-13

**Authors:** 


### HOSPITAL SUNDAY FUND.

### MEETING AT THE MANSION HOUSE.

SPEECHES BY THE ARCHBISHOP OF CANTERBURY, SIR DOUGLAS POWELL, AND SIR FREDERICK TREVES.

A LARGE and influentially-attended meeting was held at the Mansion House on Monday (8th June), under the presidency of the Lord Major, in connection with the Hospital Sunday Fund, "to further the interest in our hospitals and medical charities."

In opening the proceedings the Lord Mayor said they were met primarily to advocate the claims of the Hospital Sunday Fund and he was pleased to be able to announce that they had already made a most excellent beginning for this year. £4,300 had been received as a result of the previous day's collection in St. Paul's Cathedral. (Applause.) How greatly this result was owing to the action of the King and Queen only those closely associated with the Fund could know, and he sincerely hoped and believed that the noble example set by their Majesties in taking a personal interest in the Fund would permeate the whole of the congregations to whom they looked next Sunday, so that their Majesties might have the gratification of knowing that their effort had not been in vain and that the collection for the Fund this year had proved a record one.

THE ARCHBISHOP OF CANTERBURY.

The Archbishop said that one who spoke so often as an Archbishop was frequently confronted with the difficulty that his audience was totally unfamiliar with the subject on which he was addressing them. That day, however, the opposite was the case; the audience was already so conversant with the facts which had brought them together that he felt it to be almost an impertinence to advocate the claims of the hospitals before them. But they wanted to emphasise the fact that their corporate life was not an empty term, but meant what it said. And further, they desired to emphasise the fact that those who deliberately set aside the claims of the London hospitals for real, substantial, and permanent support, were placing themselves outside the pale of citizenship altogether. So definite was the call upon them all, so real were the needs, that he simply failed to understand the attitude of those who, while claiming the ordinary position of our common life, did not answer to that call. He knew of no other way in which this fundamental responsibility—the answerableness of the strong for the care of the weak—could be shown than by supporting the Fund that brought them together that day. Hospitals were the distinguishing mark which set apart Christendom from the civilisations that had preceded it, with the marked exception of the land which gave it birth. For centuries chivalry was the characteristic of our nation's life, and to-day no one liked to be called unchivalrous. Chivalry had been defined as the reverence of strength for weakness, and they were showing that reverence as they pleaded the cause of the hospitals that day. Nearly 400 years ago Sir Thomas More fashioned his wonderful ideal of what a nation's life ought to be. His Utopia was a great thinker's idea of the right and proper way in which a nation with great cities should carry on its work, and many of them might remember the emphasis he laid on the part that hospitals must take in the life of a great city.

"In the circuit of the City," he wrote, "a little without the walls, they have four hospitals, so big, so wide, so ample, and so large that they may seem four little towns, which were devised of that bigness to the intent the sick, be they never so many in number should not be too thronged or strait. . . . These hospitals be so well appointed and furnished to work all things necessary to health, and moreover so diligent attendance through the continual presence of cunning physicians is given that though no man be sent thither against his will, yet notwithstanding there is no sick person in all the city that had not rather lie there than at home in his own house."

They had statistics to show how far short they fell, in London, four centuries later, from that ideal set forth in Tudor times, as regards either the amplitude of their supply or the funds for its maintenance. Happily, in one respect they had realised that ideal, for they would find it hard to mend the "diligent attendance through the continual presence of cunning physicians." And moreover they had devised what Sir Thomas More never dreamed of in that great compliment to the physicians, namely the devoted band of nurses. He wondered whether they fully recognised all that was implied in the skill and competence of the thousands of devoted women who had learned the art of nursing within the hospital walls. Not long since he had had occasion to look over the rules of one of the great hospitals, and among those laid down for the staff of a hundred years ago and still extant, though not current, was one which set forth that it was strictly against orders for a nurse who came in from a tavern late at night to put a patient out of bed in order to occupy it herself! (Laughter). If they wanted to measure the distance they had travelled in a hundred years, they had only to think of what was implied in the necessity for such a rule as that. If it were true that they were so painfully inadequate as to the number of beds, or the funds required to keep them constantly full, they could only ask, and ask again, what was the reason they should have to go through the annual process of entreating those who could to do so obvious a work? It was not because the people who knew did not care, but because those who ought to know did not, and because they could not get them to know. That that was so was made plain by the object lesson of the war, when, as soon as it was made clear that there was need for a full and adequate supply of what was necessary for the care of the sick, funds were not lacking, and if they had wanted them they could have had the necessary funds ten times over. But in the ordinary round of life, those things which came home to them in the poetry and tragedy of a great war, slipped out of sight. If it were only realised that London was crying out to-day, the miserable amount of £50,000 or £60,000 which the Sunday Fund provided every year would be provided ten times over in a week. It must be that a vast number of people let the whole subject slip past them, and if the result was to be different in the future it must be brought home to them. It would be graceless and thankless not to recognise how men of great wealth had given with no ungrudging hand in some specific way or in the quiet, regular maintenance of some important part of our hospital system. But when all allowance had been made for this, and they compared London's wealth with London's needs and the amount of support given to the hospitals, they could only bow their heads with shame. The object of that meeting was not to persuade those present to give, for by their presence they showed they needed no persuasion, but to get them to make appeals to all their friends and acquaintances. Of course, all begging was unpleasant, but he hoped and trusted that the outcome of that meeting would be that hundreds of those who gave themselves would cease to be content with that, but would persuade others also, and so they would have a greater recognition on the part of the public at large of the claims which those invaluable institutions had upon their corporate life. He moved the resolution entrusted to him: "That, in view of the great interest taken by their Majesties in promoting the welfare of the Metropolitan hospitals, this meeting pledges itself to use every effort to make the Hospital Sunday collection a record one this year." (Cheers.)

Sir Douglas Powell, in seconding the resolution, said that if there could be any question as to the basis of the claims of that Fund on popular consideration, that question would be amply answered by reference to the support and sympathy and persistent aid which the King had afforded to the Fund since its very foundation—support which had not been lessened one whit since the great increase in care to which his Majesty had recently been called. The King was so well informed on all matters connected with the charities of his kingdom, that the very fact of the Fund having for so long met with his approval and support was strong evidence of its worthiness. Again, there was attached to it a very strong committee of prominent and well-known men who devoted skill and time to the distribution of the Fund; and amongst the laws of the Constitution there was the rule that any institution desirous of being recognised by the Fund must have a properly constituted committee, and present a yearly report and a balance-sheet audited by public auditors for three years preceding any grant from the Fund. Another rule provided that such institutions should be recognised as having special merit which provided an officer for the purpose of noting that the Charity was not abused by those in such circumstances that they ought not to apply for help. They looked to Mr. Loch, of the Charity Organisation Society, to help them in this matter of abuse; but he thought Mr. Loch, in an able article he had written, had dealt somewhat ungenerously with medical officers of hospitals in expecting that they should investigate the circumstances of the patients. It was their business to deal with the illnesses of their patients and not with their circumstances. As to the circumstances which underlay and led to illnesses, he urged that these should be looked to by the sanitary authorities, who might be sure they would receive all possible aid from the physicians. Sir Douglas next dwelt on the educational value of hospitals to their patients. No man could enter the wards of a hospital without being permeated with the spirit of order and cleanliness that prevailed, nor could he fail to learn something of self-discipline and self-respect, and also of sympathy with his fellow-sufferers. He passed out again primed with these as with a ferment, and could not help being an influence for good on those among whom he went. Those who helped the hospitals had the satisfaction of knowing that they were doing the best thing possible for the suffering poor, and they had this further advantage of knowing, when their own hour of need came, that the hospitals were fertile centres of advancing medical science and quickened medical thought. Cases were thought out and formed a subject of discussion and interest to all concerned. Knowledge was thus spread throughout the country to medical men of all districts, and the public had the advantage of that increased science and art of medicine, vitalised from these great centres. Undoubtedly hospital expenditure was increasing and must increase. They had increased nursing power, and medical appliances, extensions of departments, and installations of electrical and bacteriological appliances. One of the hospitals to which he had the honour of being connected had spent no less than £1,200 on electrical and bacteriological departments recently, but this outlay had given a tenfold return to the public in the advancement of medical science and the aids to treatment which it had brought about. With this increase in the cost of hospitals and appliances better and quicker results were obtained for the patients. He could recall the time when many diseases, medical and surgical, but particularly the latter, used to occupy the beds for six, eight, and twelve weeks; now the same cases were discharged in one-half, one-third, and even one-quarter of that time. (Cheers.) Therefore, although the annual expense per bed might be increasing, the cost per patient, owing to the larger number passed through the same number of beds, was by no means so large, so that the increased amount spent afforded more relief to a greater number of people—they had less pain and a quicker return to wage-earning power. One way of lessening expense was by a decreased use of stimulants. The amount of stimulants given 20 years ago was greatly in excess of that which more recent experience had led them to recognise as useful, and to-day alcoholic stimulants were ordered definitely, and definitely prescribed by physicians, and their effects were jealously watched throughout the time the patient remained under treatment. Hundreds of pounds yearly were saved to the hospitals by this watchfulness on the part of those in authority. (Cheers.) It might be said that if these institutions were of such public value beyond their mere charitable objects, why not make them rate-supported? Why, it might be asked, should busy, hard-working, and generous people have all this anxiety and trouble to raise funds to keep them going? He was aware of reasons which might be urged in favour of such a change, but if hospitals were placed on a business footing, in such a way, the quality of love and mercy now associated with them—that priceless impulse to give to a deserving cause—would vanish, and that would, indeed, in his opinion, be a great loss. Let them still endeavour to go on rendering those deeds of mercy to the sick poor; and he trusted that the splendid advocacy which many of them heard in St. Paul's on Sunday would be repeated throughout London and the country next Sunday, and that the response would be so liberal as to render quite unnecessary any suggestion for placing our hospitals and medical charities on the rates. (Cheers.)

### Sir Frederick Treves.

Sir Frederick Treves, in supporting the resolution, said that after the two admirable speeches to which they had listened it was scarcely necessary for him to say a word in advocacy of the claims of the hospitals, but he would refer to two matters not touched upon so far. The first was that they scarcely recognised to what an enormous degree during the last 25 years the work of the hospitals had been extended. They scarcely recognised that within 20 years the whole treatment in both medicine and surgery had been revolutionised. Twenty-five years ago a hospital was little more than a place in which the poor man could find shelter when he was ill or injured. The hospital was now an absolutely necessary institution to carry out treatment which 25 years ago was unknown. He mentioned hypodermic injection, serum treatment, treatment by baths, treatment based on precise chemical calculation and observation, the use of the *x*-rays, the Finsen light, and, above all, operation. The extent of the increase in such directions had been almost incredible. Twenty years ago the London Hospital possessed only one operating theatre, used only one day a week. It had now five, occupied every day in the week. The hospitals now appealed to an enormously increased section of the public, and no longer simply to the poor. So elaborate were some of the operations that they could only be carried out in hospitals or in the houses of the very well-to-do. They had now to admit many into hospitals who would be indignant if called poor, but who were totally unable to pay for the expense of such methods as were now necessary. Broken limbs, for instance, should now always be set under the *x*-rays, but it could not be done in a cottage; and all these methods of treatment were such that patients now flocked to hospitals and were admitted, and rightly so, who would never have dreamed formerly of coming within their walls. Hospitals 25 years ago were not popular places. People had a dread, and not without reason, of the hospital. Now that was no longer the case. He pressed upon them that they were doing good to an enormously increased section of the community, and doing a kindly charity to those who might be said to represent the backbone of the country. (Hear, hear.) The question might be asked—and this was his other point—What did the charitable public get in return for all their magnificent gifts to these hospitals? They obtained more than was imagined: they obtained so much that he did not hesitate to say the debt was paid many times over. In this country all medical education lay in the voluntary hospitals; there was no State-supported system. The efficiency of the medical men who were in practice in the present day and those who would be in practice in the future depended upon the efficiency of the great voluntary hospitals. As soon as the efficiency of those hospitals was curtailed for lack of funds, so soon would they curtail the efficiency of their medical men. If they wished to have the medical men of this country well equipped for their profession, they could not better forward that object than by supporting the voluntary hospitals. Practically all research was carried out by private persons in voluntary hospitals, and at their own expense. They were all eager with excitement and hope for the discovery of a cure for cancer. Could he represent the value of that in any line of figures, no matter how long? It would be discovered by some private person in some voluntary hospital, and be presented to the world absolutely free. (Hear, hear.) They might say that had not yet come; but one thing as big had come. Could they express in any coin the value to the world of the gift of antisepticism, not merely in saving pain, but in saving life (hear, hear). It had rendered possible methods of treatment which were only a dream a generation ago. That was a free gift to the world by a man who discovered it by researches conducted at his own expense in a voluntary hospital, and he was sure that Lord Lister would himself say that without the voluntary hospitals and the liberal attitude which their governing bodies had always shown towards research, antisepticism would still be unknown to the world. Those who had been most generous in supporting these hospitals had, therefore, had some return for their generosity. It was said that this matter was so enormous, it affected the wage-earning people to such a degree, that State aid was necessary. They already had rate-supported hospitals and

Military and Naval hospitals supported by the State, and he did not think they provided any argument for hospitals supported in that manner. If they made any comparisons they were not in favour of the State-supported hospitals. (Hear, hear.) He reminded them that the hospital was a guest-house in which the poor man was entertained by the rich. That magnificent work represented the kindly hand of the rich held out to the poor. No one could say that there was anything in the history of the British race grander than this enormous hospital system supported by voluntary aid. No country could present such a picture, and if they took away that element they would have done infinite damage. No person who had done other than mixed with the hospitals knew anything of the almost tragical gratitude of poor people for the care and attention which was given them. As an illustration of this he would tell them the story of a coin he held in his hand, which represented wealth no person could purchase. Many years ago a Norseman, a sailor, came to the London Hospital and came under his care, and after a time left the hospital cured, and able to follow his employment. The matter slipped from his memory, but three weeks afterwards the man came to his house. He had no idea he knew his name, much less where he lived. In a speech no courtier could have excelled in affection and gentleness he begged him to accept the coin. It was a 20-krone piece, worth some 15s. When he left home three years before, his wife had sewn it in his belt and charged him not to touch it till he was starving. "I am proud to say," he said, "that I have been starving since I left the hospital. I was so determined you should have this money that has been between me and death this three years that I have been starving. I have been hunting for a ship, I have found one. Please take this coin." And, said Sir Frederick in conclusion, that coin represents the sort of wealth that is beyond the dreams of avarice. It is like the gold of which it was said "and the gold of that land is good." (Loud cheers.)

Sir William Church moved, and Monsignor Stanley seconded, a vote of thanks to the Lord Mayor for the use of the Mansion House, which the Lord Mayor acknowledged, and the proceedings came to a close.

